# Dynamic network features of functional and structural brain networks support visual working memory in aging adults

**DOI:** 10.1162/IMAG.a.5

**Published:** 2025-05-22

**Authors:** Josh Neudorf, Kelly Shen, Anthony R. McIntosh

**Affiliations:** Institute for Neuroscience and Neurotechnology, Simon Fraser University, Burnaby, Canada; Department of Biomedical Physiology and Kinesiology, Faculty of Science, Simon Fraser University, Burnaby, Canada

**Keywords:** resting-state functional magnetic resonance imaging, dynamic functional connectivity, diffusion-weighted magnetic resonance imaging, network control theory, healthy aging, visual working memory

## Abstract

In this work, we investigated how the relationship between structural connectivity and the dynamics of functional connectivity changes with age to benefit cognitive ability. Visual working memory (VWM) is an important brain function that allows us to maintain a mental representation of the world around us, but its capacity and precision peak by around 20 years old and decrease steadily throughout the rest of our lives. This research examined the functional brain network dynamics associated with VWM throughout the lifespan and found that Default Mode Network and Fronto-Parietal Network states were more well represented in individuals with better VWM. Furthermore, transitions from the Visual/Somatomotor Network state to the Attention Network state were more well represented in older adults, and a network control theory simulation demonstrated that structural connectivity differences supporting this transition were associated with better VWM, especially in middle-aged individuals. The structural connectivity of regions from all states was important for supporting this transition in younger adults, while regions within the Visual/Somatomotor and Attention Network states were more important in older adults. These findings demonstrate that structural connectivity supports flexible, functional dynamics that allow for better VWM with age and may lead to important interventions to uphold healthy VWM throughout the lifespan.

## 1 Introduction

Visual working memory (VWM) represents one of the earliest aspects of cognitive ability to decline over a lifespan, with decreasing capacity and precision starting at around 20 years of age that continues through middle and old age. In fact, by middle age VWM ability is indistinguishable from that of 8 to 9 year olds (Brockmole & Logie, 2013). For this reason, understanding how VWM is supported by the brain could benefit all adults regardless of their current stage in the lifespan. Understanding how brain adaptations stave off this decline could lead to interventions that support these adaptations as early as the beginning of adulthood and provide long-term benefits to cognitive health later in life.

VWM allows individuals to remember and mentally manipulate visual information over short time scales, and as such represents a foundational building block for more complex cognitive tasks relying on a mental representation of the world around us, including navigation and spatial problem solving. VWM utilizes multiple systems in the brain that are important for visual processing of color and shape information, attentional orienting, and working memory encoding, maintenance, and retrieval. Working memory (WM), in general, engages a frontoparietal network of brain regions, including dorsolateral prefrontal cortex, posterior parietal cortex, and presupplementary motor areas (D’Esposito, 2007;Smith & Jonides, 1999;Wager & Smith, 2003). VWM specifically relies on visual processing regions of the occipital cortex (Harrison & Tong, 2009), and the posterior parietal cortex has been associated with spatial reasoning and attentional processing, becoming more active as the number of items maintained increases to the individual’s WM limit (Todd & Marois, 2004; seeSchurgin, 2018for a review). Dynamic network analyses that consider how different brain networks are utilized over time have demonstrated that WM tasks reduce the overall modularity in the brain compared to rest, resulting in brain networks that communicate with one another more, while WM training produces more segregated default mode and task positive (i.e., dorsal attention) networks (Bassett et al., 2015;Finc et al., 2020). Network control theory analyses of brain function during a WM task have identified that signaling between the salience network (i.e., ventral attention network), frontoparietal network, and default mode networks predicts task performance, with the anterior insula and dorsolateral prefrontal cortex acting as important regions facilitating this signalling (Cai et al., 2021). This research makes clear that VWM represents a prime example of a cognitive ability that requires efficient brain network configuration and the ability to engage multiple networks dynamically, in a coordinated fashion. Further understanding of the functional brain network dynamics and structural connections supporting these dynamics is needed, especially in the context of how specific subnetworks change the way they support VWM across the lifespan.

The healthy brain constantly adapts as context and task demands change, and even during rest. This adaptation can be described as motion through a high-dimensional space of possible brain states. For example, in one moment the brain can occupy a state wherein the default mode network is activated, often associated with mind wandering and more frequently occurring during rest, while in the next moment the brain can switch states to make use of the frontoparietal network, which is more consistently activated during demanding tasks (e.g.,Cornblath et al., 2020). Of particular interest is the question of how the transitions between one brain state and another may be unique across individuals and tell us something about that person. How is a person’s unique pattern of transitions between brain states related to the brain’s structure, and other features that set individuals apart (e.g., age and cognitive ability)?

These brain states, separable across time and relying on different combinations of brain regions, can be extracted from brain activation signals using modeling methods including Hidden Markov Modelling (HMM;Haussler et al., 1992) and Leading Eigenvector Dynamics Analysis (LEiDA;Cabral et al., 2017). HMM was initially developed for applications to protein and DNA sequencing, and has been used widely for modeling data that follows a sequence, while LEiDA, developed for neuroimaging data, accomplishes similar goals while integrating a step that extracts the leading eigenvector, capturing dominant connectivity patterns while diminishing the effects of noise. Both of these approaches have been applied to neuroimaging data, revealing distinct brain states at different points in time relying on separable combinations of regions (e.g.,Cabral et al., 2017;Faber et al., 2023,^2024^;Vidaurre et al., 2017). Important questions arise from findings that the brain coordinates the function of different combinations of regions depending on when you observe its activity. Namely, how can the brain produce multiple repertoires of functional synchronization when the structural connections (white matter tracts of axon bundles) between regions are static at short timescales? This question has been investigated previously (e.g.,Deco et al., 2011;Honey et al., 2009), and recent research using network control theory (NCT) has demonstrated that the structural connectivity network of the brain is organized to afford transitions between states via efficient stimulation to certain regions of the brain that produce cascading signals and push brain activity into new states (Gu et al., 2015,^2017^;Kim & Bassett, 2020;Lynn & Bassett, 2019;Parkes et al., 2023). This work has demonstrated that although brain function can vary widely over time, the structural connectivity does constrain to what extent certain functional states are possible.

On the one hand, NCT has allowed for interesting simulation-based predictions about how structural connectivity constrains network dynamics, and on the other hand, a number of modeling approaches including HMM and LEiDA allow for the data-driven extraction of network states from real-world time series data such as resting-state fMRI. However, the comparison of these structural and functional perspectives of brain dynamics remains a needed research direction. Furthermore, investigating how the dynamics of the structural and functional brain networks differ across stages of adulthood, and how these differences either support or hinder the brain’s ability to perform important cognitive tasks, will give us a better sense of which features of structural and functional brain dynamics are beneficial rather than detrimental. A developmental study of children and adolescents has investigated how functional dynamics change between rest and a working memory (n-back) task, and how structural connectivity constrains functional brain state transitions under these conditions (Cornblath et al., 2020). This research found that FPN state persistence increased during task compared to rest, and that the rate of occurrence of the DMN state was greater during rest than task. Patterns of state transitions were associated with WM task performance, and the overall amount of energy needed by the structural connectivity network to produce a state change was associated with age and working memory performance. An important next step is to extend this work to adulthood and older age to determine whether functional dynamics during rest can indicate trait differences in VWM, and whether this relationship changes with age. Furthermore, it remains to be demonstrated whether the regions used by the structural connectivity network to support efficient transitions between these states change across the lifespan, and how these changes relate to VWM.

Although age is commonly associated with declining cognitive ability and negative brain changes, we have used multiscale entropy to demonstrate that some changes in the complexity of functional activity with age are associated with spared cognitive ability (Heisz et al., 2015). Furthermore, we have found that certain aspects of structural brain network reorganizations were associated with both declining as well as spared cognitive ability in older adults (Neudorf et al., 2024). In particular, increased local interhemispheric connections and specific regional differences in the organization of hub regions were associated with spared cognitive ability (Neudorf et al., 2024).

For the current work, we identified the dynamic connectivity patterns (brain states) from both functional and structural brain network perspectives, investigated how the time spent in these states and the pattern of transitions between states differ across the lifespan from younger to older adulthood, and whether some of these differences contribute to better VWM in older adulthood. Recent research has demonstrated strong coupling between structural and functional networks in the brain (Benkarim et al., 2022;Neudorf et al., 2022;Sarwar et al., 2021;Schirner et al., 2018), that this coupling is altered across the lifespan (Zimmermann et al., 2016), and that the structural network constrains the range of functional dynamics possible with an individual’s brain network (Gu et al., 2015;Lynn & Bassett, 2019). For these reasons, we will examine how differences in structural connectivity with age may support specific functional dynamics to preserve VWM.

## 2 Methods

Data came from the Cambridge Centre for Ageing and Neuroscience (Cam-CAN;Shafto et al., 2014) dataset. Data collection followed the Helsinki Declaration, and was approved by the local ethics committee, Cambridgeshire 2 Research Ethics Committee (reference: 10/H0308/50). The full sample of subjects with neuroimaging data included 653 subjects. Participant ages ranged from 18.50 to 88.92 (mean = 54.825,*SD*= 18.593). Younger adult ages ranged from 18.50 to 49.92 (mean = 36.420,*SD*= 8.495,*N*= 279, 146 female, 133 male). Older adult ages ranged from 50.17 to 88.92 (mean = 68.593,*SD*= 10.338,*N*= 373, 184 female, 189 male). A single participant was missing age information. The resting-state functional MRI (rs-fMRI) subsample included 197 subjects that passed our quality control criteria (see below). Participant ages ranged from 18.50 to 88.92 (mean = 48.310,*SD*= 17.388). Younger adult ages ranged from 18.50 to 49.83 (mean = 35.471,*SD*= 8.176,*N*= 114, 67 female, 47 male). Older adult ages ranged from 51.92 to 86.08 (mean = 65.945,*SD*= 9.308,*N*= 83, 43 female, 40 male). The diffusion-weighted MRI (dMRI) subsample included 594 subjects that passed our quality control criteria (see below). Participant ages ranged from 18.50 to 88.92 (mean = 55.414,*SD*= 18.090). Younger adults (YA; age < 50) ranged from 18.50 to 49.92 years (mean = 36.966,*SD*= 8.385,*N*= 244, 131 female, 113 male), and older adults (OA; age > 50) ranged from 50.17 to 88.92 years (mean = 68.275,*SD*= 10.163,*N*= 350, 170 female, 180 male). An age of 50 was used to split the participants into YA and OA groups as the age of 50 corresponds to approximately the age by which the VWM of a middle-aged individual declines to the level of a child (aged 8–9 years old) based on past research (Brockmole & Logie, 2013). Furthermore, after this point VWM continues to decline, suggesting that there may be a meaningfully different relationship between brain and VWM than had been previously experienced in the lifespan. This age split was also convenient as it roughly equated the age range between the groups.

### 2.1 Structural MRI

The T1-weighted Magnetization Prepared RApid Gradient Echo (MPRAGE) sequence was performed using a repetition time (TR) of 2250 ms and echo time (TE) of 2.99 ms, with a flip angle of 9°, field of view (FOV) of 256 × 240 × 192 mm, and 1 × 1 × 1 mm voxel size. The T2-weighted sampling perfection with application-optimized contrasts using different flip angle evolution (SPACE) sequence was performed using a TR of 2800 ms, a TE of 408 ms, an FOV of 256 × 256 × 192 mm, and 1 × 1 × 1 mm voxel size.

### 2.2 Functional MRI

The rs-fMRI sequence was performed using a Gradient-Echo Echo-Planar Imaging (EPI) sequence with a TR of 1970 ms, TE of 30 ms, flip angle of 78°, FOV of 192 × 192 mm, and 3 × 3 × 4.44 mm voxel size. This sequence acquired a total of 261 volumes over 8 min and 40 s, with each volume containing 32 axial slices. The preprocessing of these data relied on TheVirtualBrain-UK Biobank pipeline (Frazier-Logue et al., 2022), which has updated the FMRIB Software Library (*FSL*;Jenkinson et al., 2012) based UK Biobank pipeline (Littlejohns et al., 2020) to account for issues that can occur due to atrophy in aging brains using quality control methods to minimize artifacts (Lutkenhoff et al., 2014). This pipeline also outputs parcellation-based blood oxygen level dependent (BOLD) time-series data which were used for the dynamic functional connectivity analyses. These time-series data were bandpass filtered to include frequencies between 0.1 and 0.01 Hz.

Using 41 imaging-derived phenotypes (IDPs) related to the T1w and T2w structural image quality, rs-fMRI imaging quality, and structural-functional registration from the TVB UKBB pipeline (Frazier-Logue et al., 2022) as predictor variables and human rated scores of rs-fMRI quality (based on visual assessment of the fMRI fieldmaps, motion, registration,*FSL MELODIC*independent component labeling accuracy, functional connectivity matrix, and time-series carpet plot) on a scale of 1-5 (1 is excellent, 2 is good, 4 is poor, and 5 is very poor) as the criterion variable, a random forest regression machine-learning approach was trained to predict rs-fMRI quality on a subset of the Cam-CAN data (140 participants). The*auto-sklearn*(Feurer et al., 2015) Python library was used to aid selection the best performing machine-learning algorithm and parameters, and Random Forest Regression was selected (*scikit-learn*;Pedregosa et al., 2011). Human rated quality was rescaled to the range of 0 to 1, with an original score of 1 corresponding to 0.1 and with 0.2 increments between scores. When applying this model to the unrated subjects (*N*= 499), a score less than 0.4 corresponded to a passing value, a score greater than 0.6 corresponded to a failing value, and scores between 0.4 and 0.6 were selected for manual human rating. Using this same procedure with the manually human rated subjects (*N*= 140), in a K-fold validation scheme (K = 5) repeated over 100 iterations, setting aside the subjects in the medium range of 0.4 to 0.6 and looking only at those subjects identified confidently as good or bad we observed a false negative detection of a bad result (falsely indicating the result was good) in a mean number of 4.990 subjects (standard deviation;*SD*= 1.396) out of the 63 empirically good results (7.9% false negative; 92.1% accuracy), and a false positive detection of a bad result (falsely indicating the result was bad) in a mean number of 6.990 subjects (*SD*= 1.179) out of the 77 empirically bad results (9.1% false positive; 90.9% accuracy). When applying the trained model to the unrated subjects’ data, we identified 143 subjects with good results, 206 subjects with bad results, and 150 subjects with results selected for manual human rating. The manual human rating of the remaining subjects resulted in 10 more good results, for a total of 216. Out of these results, 197 had corresponding structural connectivity, demographic, and behavioral measures of interest and were therefore retained. No subjects were included that had more than 5 timepoints with framewise displacement of more than 1 mm. Most subjects had only one or no timepoints above 0.3 mm and all timepoints less than 1 mm, as a function of the numerous other factors mentioned that were considered in order to accept only high-quality data (only the top 30.8% of subjects were included).

### 2.3 Diffusion-weighted MRI

The dMRI imaging was performed using a twice-refocused sequence with a TR of 9100 ms, TE of 104 ms, FOV of 192 × 192 mm, and voxel size of 2 × 2 × 2 mm, with 30 directions of 66 axial slices having a b-value of 1000, 30 directions of 66 axial slices having a b-value of 2000, and 3 images of 66 axial slices having a b-value of 0. The structural connectivity (SC) measures of streamline probability and distance were calculated from the dMRI data using the TVB-UK Biobank pipeline (Frazier-Logue et al., 2022), which uses probabilistic tractography (*FSL bedpostx*to fit the probabilistic model and*probtrackx*to perform tractography;Hernandez-Fernandez et al., 2019;Jenkinson et al., 2012). The SC streamline probability is the number of connecting streamlines identified by the tractography divided by the total number of possible connections (i.e., normalized by the size of the region) and represents the probability of connection between all combinations of the 218 regions of interest in a combined atlas of the Schaefer 200 region atlas (Schaefer et al., 2018) and the subcortical Tian atlas (Tian et al., 2020). The subcortical regions comprised regions from the Tian Scale 1 atlas excluding the hippocampus. For the hippocampus, the Scale 3 atlas was used with the two head divisions collapsed into a single parcel. The globus pallidus was excluded due to a large number of subjects without any detectible connections to or from this region, resulting in a total of 18 subcortical regions. The SC matrices were consensus thresholded (at least 50% of participants have the connection), and participants’ data were excluded if they did not have behavioral data, had regions with no connections, or had SC density (number of non-zero connections divided by the total number of possible connections) 3*SD*or more away from the mean (retained*N*= 594; analyzed data from 197 participants with corresponding fMRI data passing quality control).

### 2.4 Visual working memory

Precision on the visual working memory task designed byZhang and Luck (2008)was used to measure VWM (seeShafto et al., 2014for more details). In this task 1 to 4 colored circles are presented peripherally to fixation and after a 900 ms delay participants are required to report the hue of the circle at the cued location. VWM declines significantly with age in this population,*R*(592) = -0.291,*p*< .001 (as demonstrated previously byBrockmole & Logie, 2013).

### 2.5 Dynamic functional connectivity

The rs-fMRI data were analyzed using a dynamic functional connectivity (dFC) analysis approach called Leading Eigenvector Dynamics Analysis (LEiDA;Cabral et al., 2017). This method uses phase coherence connectivity (e.g.,Deco et al., 2017;Deco & Kringelbach, 2016;Glerean et al., 2012;Ponce-Alvarez et al., 2015) to compute a functional connectivity (FC) matrix at each timepoint of the resting-state fMRI scan. In contrast to other dFC methods that compare the full FC matrices across timepoints, LEiDA first computes the leading eigenvector of each FC matrix, making the method less susceptible to noise and better able to detect the recurrence of a particular state. The dFC matrices are separated into distinct states by applying a clustering analysis on the leading eigenvectors for all subjects and timepoints, resulting in states that are common to all subjects. Specifically,*k*-means clustering (with*k*ranging from 2 to 20) was applied to the leading eigenvectors of all subjects’ timepoints (261 timepoints × 197 subjects = 51,417 leading eigenvectors; seeCabral et al., 2017for further details about this method). The ideal number of states was chosen based on an evaluation of the clustering analysis that maximized the Dunn’s score (Dunn, 1973), average Sihouette coefficient (Rousseeuw, 1987), and Calinski–Harabasz index (Caliński & Harabasz, 1974). The identified states were associated with one of seven Yeo Resting State Networks (Yeo et al., 2011) by matching that state’s centroid with the network having maximal overlap with the positive centroid values, as implemented by the original authors of this method (Cabral et al., 2017). With these states defined, each timepoint was then labeled according to which state the participant’s brain function was in at that moment, which allowed for calculation of the fractional occupancy (FO) of each state (probability of that state occurring at any given time) and the transition probability matrices (probability of the brain state changing from a specific state to another, or maintaining the same state, represented as a K×K matrix where K is the total number of states).

### 2.6 Network control theory

Network control theory is a method that allows for observing the constraints that a structural connectivity network exerts on the functional dynamics of that system. By assuming a linear model of diffusion for simulating how activation spreads in parallel across the network, computationally efficient calculations can be performed to minimize the total input energy and state trajectory needed to transition from an initial state to a target state. This computation is performed by minimizing the cost function involving the magnitude of the control signals and the magnitude of the state trajectory. The regions in the network that were given the largest amount of control energy were selected by this model as ideal recipients of the limited input energy. In the context of the brain, these control energy values provide insight into which regions are able to most efficiently drive a state transition given limited metabolic energy, and the utility of this method for network neuroscience has been well demonstrated in recent research (Gu et al., 2015,^2017^;Kim & Bassett, 2020). This method provides an important opportunity to investigate how the structural connectivity network may differ with age to support a state transition identified as important in the aging process via the LEiDA analysis of dynamic functional connectivity. The network control theory analysis was performed with*nctpy*(Parkes et al., 2023), using a continuous-time simulation, with a mixing parameter ρ of 1 to set equal weight to minimizing the control input energy and the neural activity, placing no constraints on which regions could be utilized for control inputs (i.e., optimal control), and using a uniform full control set allowing all regions to act as controllers with equal control over the system dynamics. Additional supplemental analyses were performed with ρ varied to verify that the results were not parameter dependent.

### 2.7 Partial least squares analysis

Multivariate partial least squares (PLS) analysis (McIntosh & Lobaugh, 2004) was used to identify latent variables (LVs), each containing weights that describe the relationship of all brain measures with age and VWM. Behavioral PLS was used to examine effects of age and VWM as continuous measures. Models including sex as a group variable were examined, but produced no significant group effects so they were not included in the reported analyses. Nuisance variable models were examined with gray matter volume for FO, TP, and network control theory, as well as mean motion for FO and TP. If these models were significant, a follow-up cosine similarity analysis was performed between the brain saliences of the nuisance variable model and the corresponding model of interest. PLS uses singular value decomposition to project the data matrix onto orthogonal LVs (similar to canonical correlation analysis). The significance of the identified LVs was determined via permutation testing. We report only the most reliable PLS weights as determined by bootstrap resampling to calculate bootstrap ratios (BSR), which are the ratios of the PLS weights (saliences) to their standard errors as determined by bootstrap resampling (Kovacevic et al., 2013;McIntosh & Lobaugh, 2004). The resampling procedures were performed using 1000 iterations for each.

## 3 Results

### 3.1 Dynamic functional connectivity

LEiDA identified five states that were occupied during the course of the resting-state fMRI scan. The first state identified was the Global Coherence state, which is described as a state in which brain regions demonstrate overall coherence in their signals (Cabral et al., 2017). The second state overlapped primarily with a set of regions commonly referred to as the Default Mode Network (DMN). The third state overlapped primarily with the ventral attention network but also included dorsal regions from the dorsal attention network and will, therefore, be referred to simply as the Attention Network state. The fourth state overlapped primarily with the Frontoparietal Network (FPN), and the fifth state overlapped with regions corresponding to both the Somatomotor Network and the Visual Network (as defined byYeo et al. (2011)functional atlas; seeFig. 1), and will be referred to as the Somatomotor/Visual Network state.

**Fig. 1.**
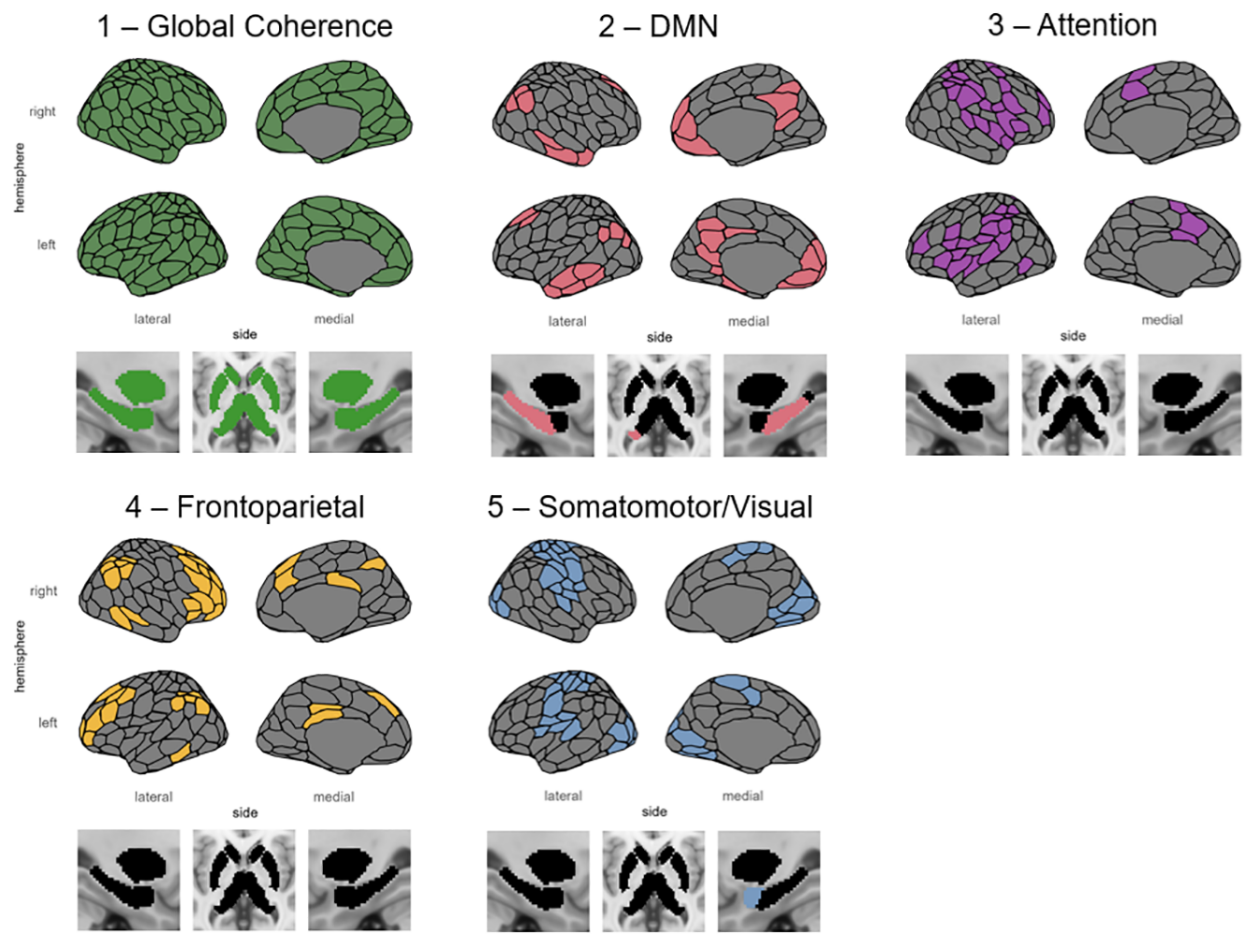
Dynamic functional connectivity states identified by the LEiDA analysis. State 1 in green represents the Global Coherence state, State 2 in pink represents the Default Mode Network (DMN) state, State 3 in purple represents the Attention Network state, State 4 in yellow represents the Frontoparietal Network (FPN) state, and State 5 in blue represents the Somatomotor/Visual Network state.

With these states defined, state timecourses were then produced for each individual, which allowed for the calculation of each individual’s fractional occupancy (FO; the percentage of time spent in each state). Behavioral PLS analyses were conducted with FO of each state as independent variables and age as the dependent variable in the first analysis, with VWM as the dependent variable in the second analysis, and with age and VWM as dependent variables in the third analysis. The first analysis with age identified a significant LV (permutation*p*= .048) that was positively associated with age, indicating that older adults spend more time in the DMN and FPN states (seeFig. 2A, B). The second analysis identified a significant LV (permutation*p*= .028) that was positively associated with VWM, indicating that less time spent in the Global Coherence state and more time spent in DMN was associated with better VWM (seeFig. 2C, D). The third analysis identified a significant LV (permutation*p*< .001) that was positively associated with both age and VWM, indicating that less time spent in the Global Coherence state and more time spent in DMN and FPN was associated with a better VWM in older age (seeFig. 2E, F). An additional mean-centered PLS was performed comparing FO in four groups: YA with low VWM, YA with high VWM, OA with low VWM, and OA with high VWM. This analysis identified 1 significant LV (permutation*p*< .001) with brainscores, indicating that it identified how FO was associated with the OA high VWM group (YA low VWM:*brainscore*[95% CI] = -.0482 [-.079 to -.017]; YA high VWM:*brainscore*[95% CI] = -.007 [-.035 to .021]; OA low VWM:*brainscore*[95% CI] = -.022 [-.050 to .006]; OA high VWM:*brainscore*[95% CI] = .076 [.039 to .114]). This effect for OA with high VWM were consistent with the behavioral PLS LV associated with increasing age and VWM, with BSRs that were reliably negative for Global Coherence and reliably positive for DMN and FPN states. The identified positive BSRs for DMN and FPN indicate that the increased time spent in DMN and FPN is not a detrimental aspect of the aging brain’s functional dynamics, but rather that this difference is advantageous for VWM. Nuisance variable models including gray matter volume and fMRI motion were not significant.

**Fig. 2.**
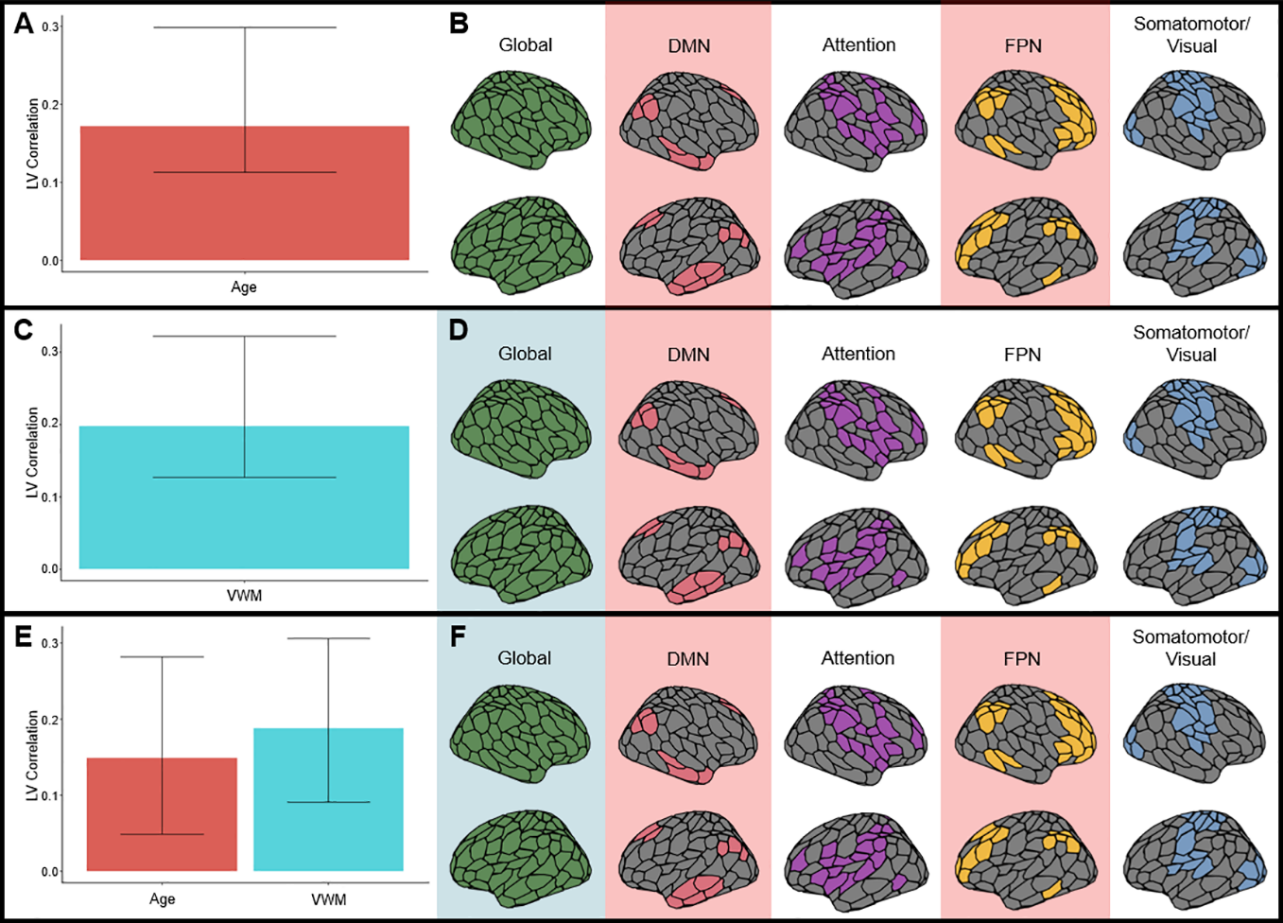
PLS analyses with independent variables of FO and dependent variable age (A, B), dependent variable VWM (C, D) and dependent variables of age and VWM (E, F). (A, C, and E) depict the behavioral correlation between the dependent variables and the LV, while (B, D, and F) represent the BSRs highlighting the reliable positive behavior-FO associations in red and the reliable negative behavior-FO associations in blue.

LEiDA also allows for the calculation of a state transition probability matrix, representing the probability of a transition occurring from state*i*in row*i*to state*j*in column*j*in the 5 × 5 matrix, where 5 is the total number of states (seeFig. 3B). Diagonal elements in the transition probability matrix represent maintenance of that state without a transition to another state. A PLS analysis with the transition probability matrix values as the independent variables and age as the dependent variable identified a significant LV (permutation*p*= .009). This LV was positively associated with age (seeFig. 3A), so that positive BSRs represent transitions (off diagonal elements) or maintenances (diagonal elements) that were more probable in older adults while negative BSRs represent transitions or maintenances that were less probable in older adults. The analysis identified the maintenance of State 2 (DMN) and State 3 (Attention) as being more prevalent in older adults, consistent with the FO analysis finding that DMN and FPN FO were greater for older adults, while adding Attention as another state that is more prevalent in older adults. The probability of transition from State 5 (Somatomotor/Visual) to State 3 (Attention) was also greater for older adults. Conversely, the probability of transition from State 2 (DMN) to State 1 (Global Coherence), from State 1 (Global Coherence) to State 4 (FPN), and from State 3 (Attention) to State 4 (FPN) were decreased in older age (seeFig. 3Bas well asFig. 3Cfor a graph representation of reliably age-related transitions and maintenances). As the only reliably age-related transition probability that increased with age, the transition from State 5 (Somatomotor/Visual) to State 3 (Attention) was investigated further from a structural connectivity perspective using the network control theory.

**Fig. 3.**
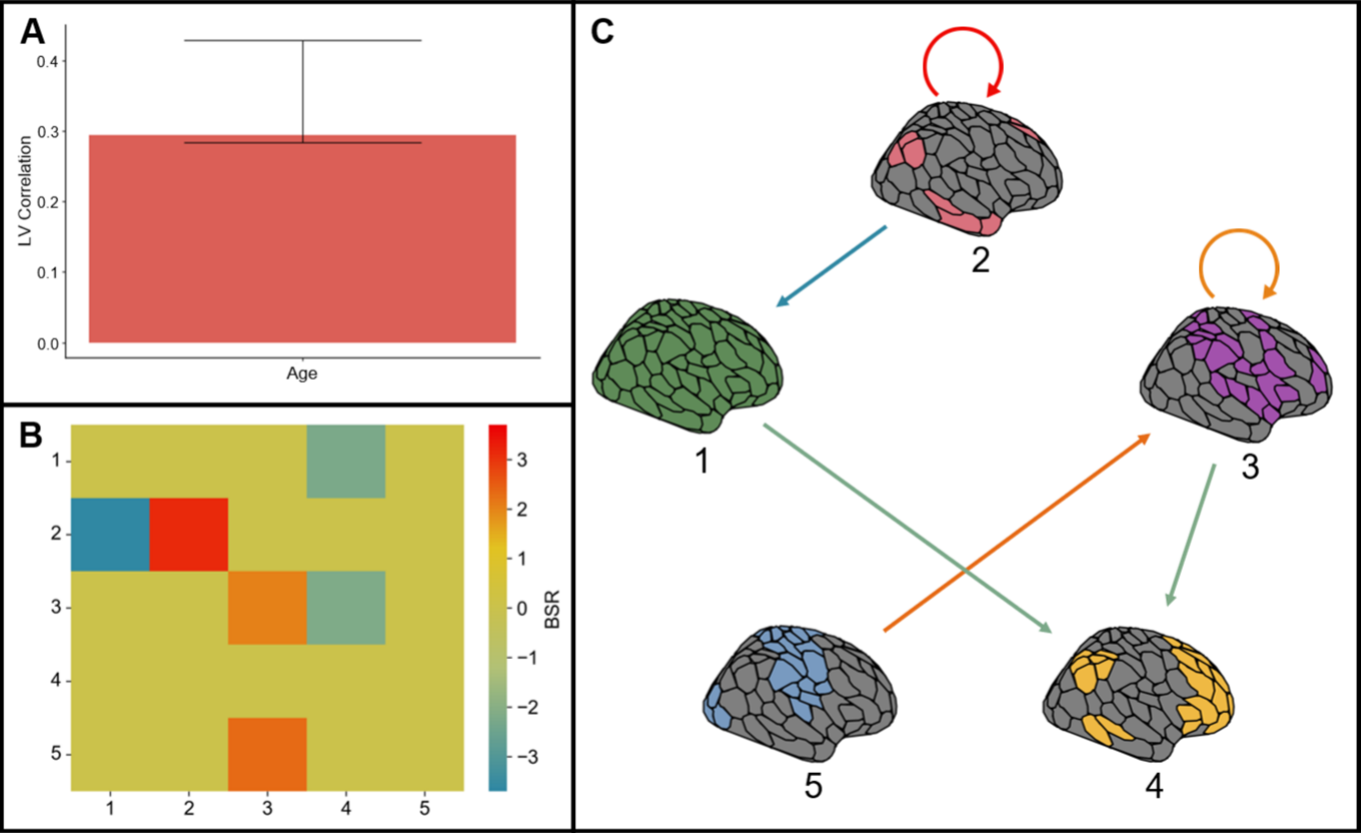
PLS analysis with independent variables of transition probabilities and dependent variable age. (A) depicts the behavioral correlation between age and the LV, while (B) and (C) represent the BSRs highlighting the reliable positive associations with age in red and the reliable negative associations with age in blue. BSRs are represented as both a transition probability matrix (B) and a transition probability graph (C).

As in the FO analysis, additional PLS analyses with VWM and Age plus VWM as independent variables were also conducted, but were not significant. The nuisance variable model including gray matter volume was not significant, but the model including fMRI motion produced a significant LV (*p*= .028). However, the cosine similarity between the brain saliences in this nuisance variable model was not significantly similar to the model of interest, indicating that these models are detecting separate effects (cosine similarity = .475, permutation*p*= .119).

### 3.2 Network control theory

**Fig. 4.**
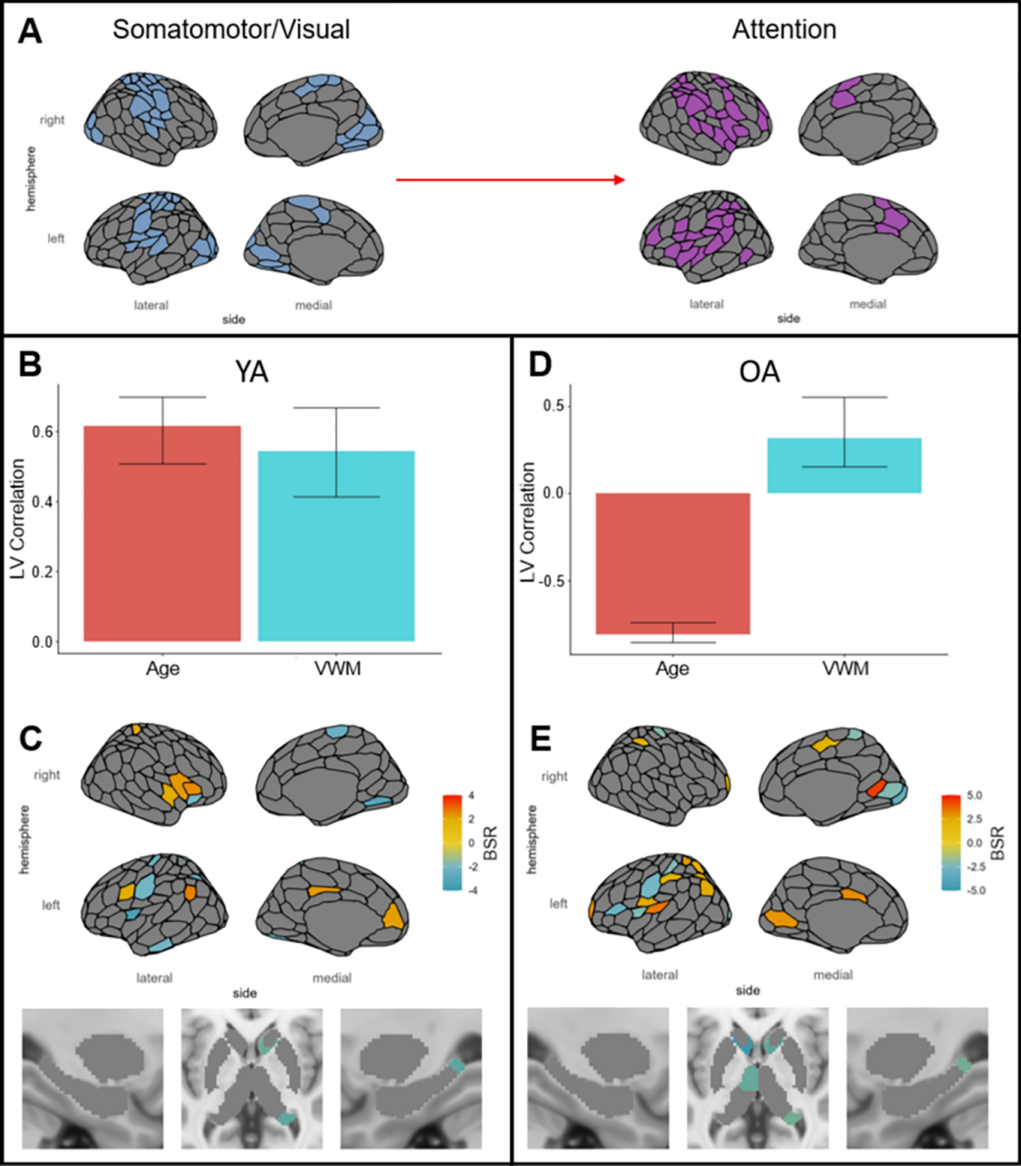
PLS analyses with independent variables of control energies for a state transition from the Somatomotor/Visual to the Attention state (A) and dependent variables of age and VWM. (B) and (D) depict the behavioral correlation of age and VWM with the LV, while (C) and (E) represent the BSRs highlighting the regions with reliable positive associations with age and VWM in yellow to red and the reliable negative associations with age and VWM in green to blue.

The network control theory analysis identified a combination of control energies given to each region corresponding to the optimal (minimized) total inputs to the structural connectivity-based simulation, resulting in a transition from State 5 (Somatomotor/Visual) to State 3 (Attention; seeFig. 4A). A high value of control energy in a region can therefore be interpreted as that region being situated in the structural network architecture in a such a way that affords an ideal level of efficient control (input energy minimized) over the transition from the Somatomotor/Visual Network state to the Attention Network state (i.e., the brain relies heavily on that region for this transition). Separate PLS analyses for YA and OA were conducted, with control energies as independent variables and age and VWM as dependent variables. The YA analysis identified a significant LV (permutation*p*= .029) positively associated with age and VWM, indicating that VWM was better with age in individuals with greater control energies in regions with positive BSRs and with lower control energies in regions with negative BSRs (seeFig. 4B). The OA analysis identified a significant LV (permutation*p*< .001) negatively associated with age and positively associated with VWM, indicating that VWM was greater for individuals on the younger end of this age range if they had greater control energies in regions with positive BSRs and with lower control energies in regions with negative BSRs (seeFig. 4D). The YA and OA analyses both identified LVs associated with better VWM, and both analyses identified an effect of age toward the age category boundary of 50 years, indicating an effect on VWM that is maximized in middle age. The regions identified in these age groups had some overlap but also clear differences. For both YA and OA the RH hippocampus tail, RH caudate, LH central sulcus, LH frontal operculum, and bilateral dorsal parietal regions were negatively associated with VWM, indicating that a reliance on these regions for the Somatomotor/Visual to Attention state transition was disadvantageous (seeFig. 4C, E). Regions unique to YA demonstrating this negative relationship between control energy and VWM included RH inferior extrastriate, RH anterior insula, and LH anterior inferior temporal lobe (seeFig. 4C). Regions unique to OA demonstrating this negative relationship between control energy and VWM included the LH anterior thalamus, LH caudate, LH ventral prefrontal cortex (PFC), RH calcarine, and RH striate cortex (seeFig. 4E). For the YA, higher control energies were associated with better VWM in a cluster of regions: the RH insula and frontal operculum, as well as an RH dorsal parietal region, LH dorsolateral and medial PFC, LH inferior parietal lobule (IPL), and LH posterior cingulate cortex (PCC; seeFig. 4C). For the OA, higher control energies were associated with better VWM in two regions of the LH insula, LH postcentral gyrus, a cluster of regions in the LH posterior parietal cortex including the IPL, SPL, and postcentral gyrus, LH striate/calcarine, LH midcingulate, RH postcentral gyrus, RH medial parietal, and RH superior extrastriate (seeFig. 4E). Nuisance variable models including gray matter volume were not significant. For an unthresholded version ofFigure 4, seeSupplementary Figure 1. Supplemental analyses with the NCT parameter ρ varied produced highly comparable PLS results, demonstrating that these results were not parameter dependent (seeSupplementary Figs. 2-5). The results of an additional PLS analysis including all ages in the same model are also available inSupplementary Figure 6.

## 4 Discussion

We have demonstrated that the theoretical capacity of the brain’s structural connectivity network to support dynamic activity is related to empirical data-driven measures of functional dynamics, that this relationship matures across the lifespan, and that elements of these dynamics support better VWM in middle age. Time spent in the DMN and FPN states increased with age and was associated with better VWM, as were decreases in Global Coherence time. Maintenance of the DMN and Attention Network states increased with age, while state transition probability from the DMN state to Global Coherence, Global Coherence to the FPN state, and the Attention Network state to the FPN state decreased with age. State transition probability from the Somatomotor/Visual Network state to the Attention Network state increased with age, and adaptations of the structural connectivity network supporting this state transition were associated with better VWM, particularly in middle age.

Our results highlight the importance of investigating*both*functional and structural brain networks from the perspective of how they support dynamics across the lifespan. Brain structure may be relatively fixed at physical and temporal macroscales, but the functions they support are dynamic. The dynamics that support better VWM with age provide an example of how the structural network informs the behavior of functional networks that support cognitive abilities. It is interesting to see that VWM was associated with signatures of brain function during rest, separate from any related or demanding task. This suggests that individuals’ patterns of functional switching in the brain and the structural connections that make the transition between the Somatomotor/Visual Network state and the Attention Network state easier represent an important indicator of VWM as a person ages. The identification of the transition between these states specifically suggests that, regardless of task demands (even at rest in this case), an individual’s ability to transition from a sensory/visual input state (more bottom-up processing) to an attention state that can exert more top-down control on these inputs translates to their ability in a VWM task that requires taking visual inputs and attending to them effectively.

It is interesting to note that the structural connectivity network control theory analysis for younger adults implicated a number of regions associated positively with VWM that overlap with the Somatomotor/Visual and Attention networks, indicating that part of the role of influencing the efficiency of these transitions lies within these networks themselves, while also implicating regions from both the DMN and FPN. This suggests that performing the state transition efficiently relies on multinetwork coordination, and that this develops toward middle age. Conversely, regions positively associated with VWM for older adults were primarily within the Somatomotor/Visual and Attention networks, suggesting an ideal multinetwork coordination is established by middle age and that age-related differences occur locally within the Somatomotor/Visual and Attention networks after middle age.

### 4.1 Connections to past research

The regions positively related to VWM in the network control theory analysis demonstrated consistency with past research, identifying the insula (RH for YA and LH for OA) as highlighted in previous network control theory research on WM task-based fMRI data (Cai et al., 2021). Furthermore, the dynamic functional connectivity analyses identified that time spent in the DMN and FPN states was not only greater in older adults, but that time spent in these states was associated with better VWM. Considering that increased segregation of the DMN should help to maintain ongoing occupation of this state, these results add to evidence from past research finding that the DMN becomes more segregated after WM training (Finc et al., 2020). Our finding that increased time spent in the FPN state at rest was associated with better VWM is also consistent with long-standing knowledge of the importance of the FPN to WM (D’Esposito, 2007;Smith & Jonides, 1999;Wager & Smith, 2003). The Global Coherence state identified by the LEiDA analysis represents a state in which brain regions are working together overall, rather than isolating a certain specialized subnetwork. It has been suggested by the authors of this analysis method that this state is related to the global signal of fMRI, which has not been definitively linked to any one cognitive process. However, past research using this method has identified the Global Coherence state as being more prominently represented in older adults with good cognitive performance compared to those with poor cognitive performance (Cabral et al., 2017). Cognitive performance in this previous research was assessed using multiple measures sensitive to executive function, memory, and mood. Interestingly, our results found that decreases in the prevalence of Global Coherence were associated with better VWM. It seems the specific domain of cognitive ability may affect the relevance of time spent in the Global Coherence state, and that, for VWM, individuals with the ability to spend more time in specific states (DMN and FPN) rather than in a Global Coherence state, and to efficiently transition from the Sensorimotor/Visual to the Attention state, are more likely to have better VWM.

### 4.2 Limitations and future directions

A longitudinal investigation utilizing this approach would provide a fuller picture of the relationship between structural connectivity, functional network dynamics, and cognitive ability. Recent research has highlighted the important differences between longitudinal and cross-sectional brain age research (Vidal-Pineiro et al., 2021). Adding a longitudinal perspective would represent an important contribution to this research investigating how the dynamic functional repertoires available are also dynamic across the lifespan, from infancy to childhood, adolescence, young adulthood, middle age, and old age. We know from both this and past research (Neudorf et al., 2024) that the network regimes that are ideal for healthy young adults are not the same as those that are ideal for older adults, so a continued exploration of brain networks across the lifespan with an eye for how these architectures address unique demands represents an important effort to diversify our understanding of how dynamic the brain network is across the lifespan.

## 5 Conclusion

An investigation of the functional and structural brain networks’ dynamics allowed us to observe age-related differences in how the brain’s structure supports its functional dynamics in a way that preserves cognitive ability. Visual working memory relies on specifically tuned brain networks in middle age that can maintain Default Mode and Frontoparietal Network states and efficiently transition from a Sensorimotor/Visual focused state to a state wherein the individual is ready to attend to important information. The functional state transition from Sensorimotor/Visual to Attention state was supported by the structural connectivity network, and different regions of the structural network were important during the approach to and the departure from middle age. This work has identified brain network signatures related to task performance based on brain structure and function at rest, suggesting that intrinsic features of brain dynamics may be valuable for assessing brain aging trajectories and relate to clinical conditions such as dementia.

## Supplementary Material

Supplementary Material

## Data Availability

Data came from the Cambridge Centre for Ageing and Neuroscience (Cam-CAN;Shafto et al., 2014) dataset, and can be accessed athttps://camcan-archive.mrc-cbu.cam.ac.uk/dataaccess. The code used for these analyses is available athttps://github.com/McIntosh-Lab/dFC_Cam-CAN.
