# Supplementary material for: Dynamic network features of functional and structural brain networks support visual working memory in aging adults
Source: Imaging Neurosci (Camb). 2025 May 22;3:IMAG.a.5. doi: 10.1162/IMAG.a.5 (PMC12319731; doi:10.1162/IMAG.a.5)
Supplement: Supplementary Material [file imag.a.5_supp.pdf]

Supplementary Materials

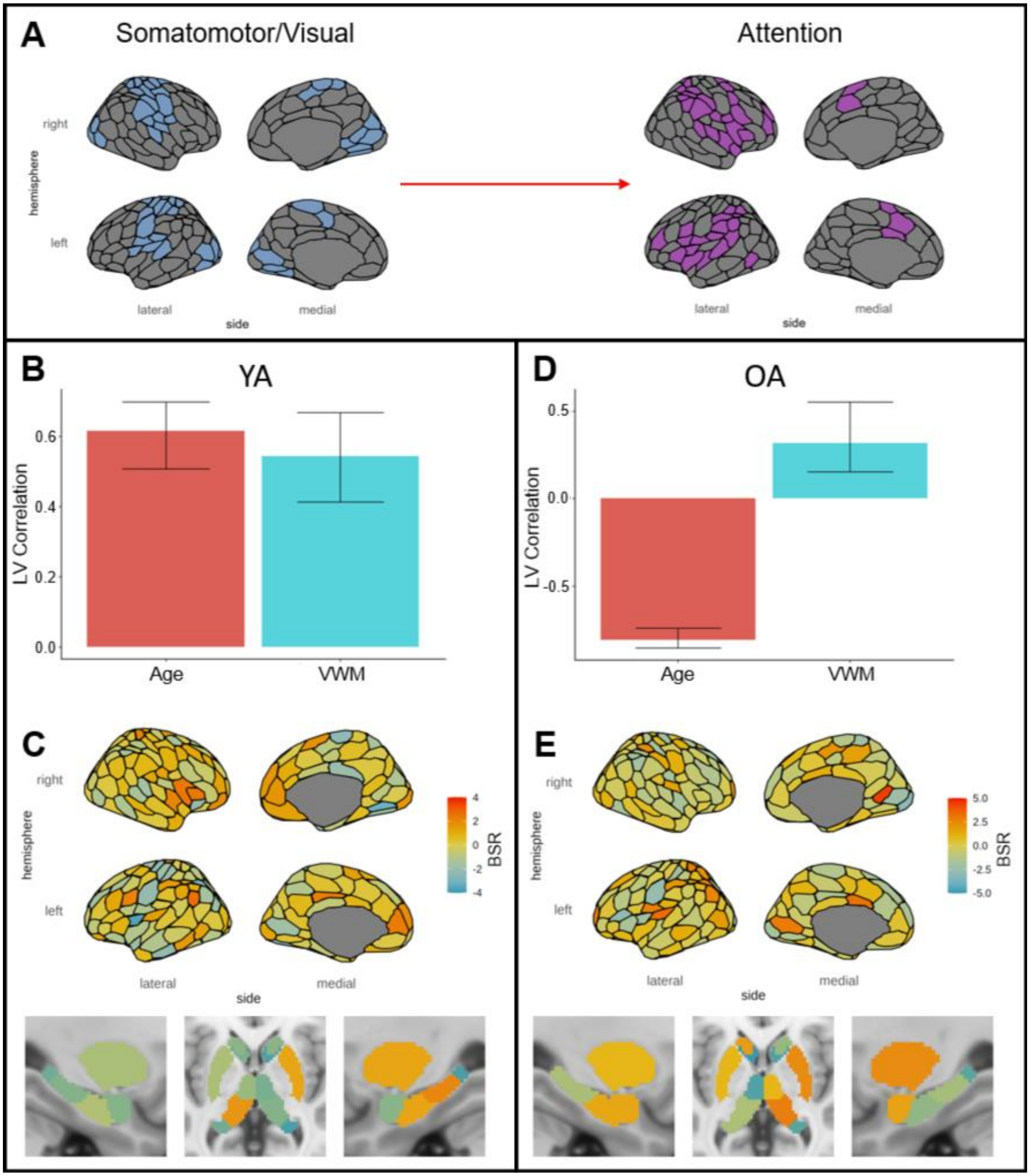

Supplementary Figure 1. PLS analyses with independent variables of control energies for a state transition from the Somatomotor/Visual to the Attention state (**A**) and dependent variables of age and VWM. (**B**) and (**D**) depict the behavioral correlation of age and VWM with the LV, while (**C**) and (**E**) represent the unthresholded BSRs highlighting the regions with positive associations with age and VWM in yellow to red and the negative associations with age and VWM in green to blue.

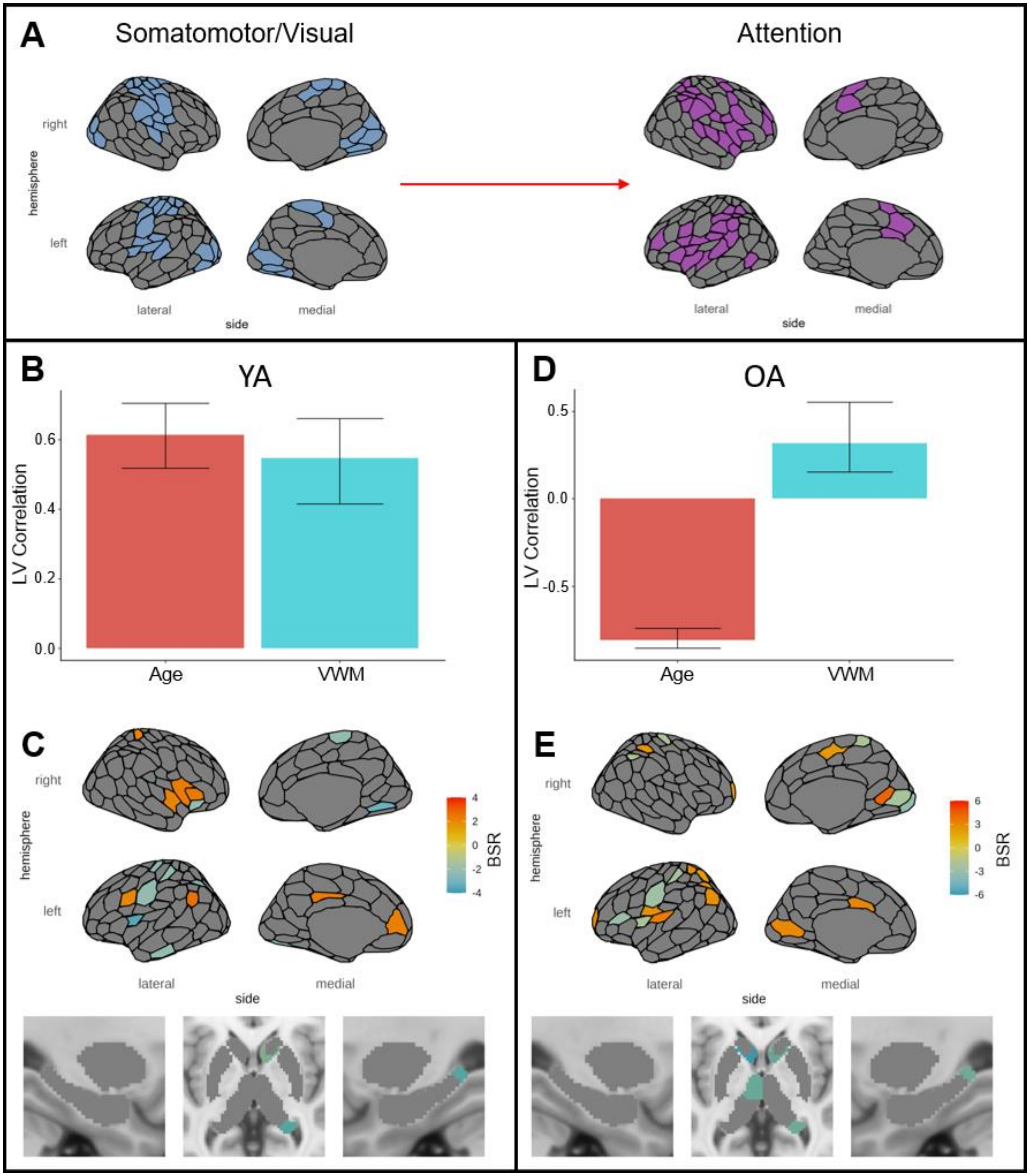

Supplementary Figure 2. PLS analyses with independent variables of control energies (with ρ = 0.9) for a state transition from the Somatomotor/Visual to the Attention state (**A**) and dependent variables of age and VWM. (**B**) and (**D**) depict the behavioral correlation of age and VWM with the LV, while (**C**) and (**E**) represent the BSRs highlighting the regions with reliable positive associations with age and VWM in yellow to red and the reliable negative associations with age and VWM in green to blue.

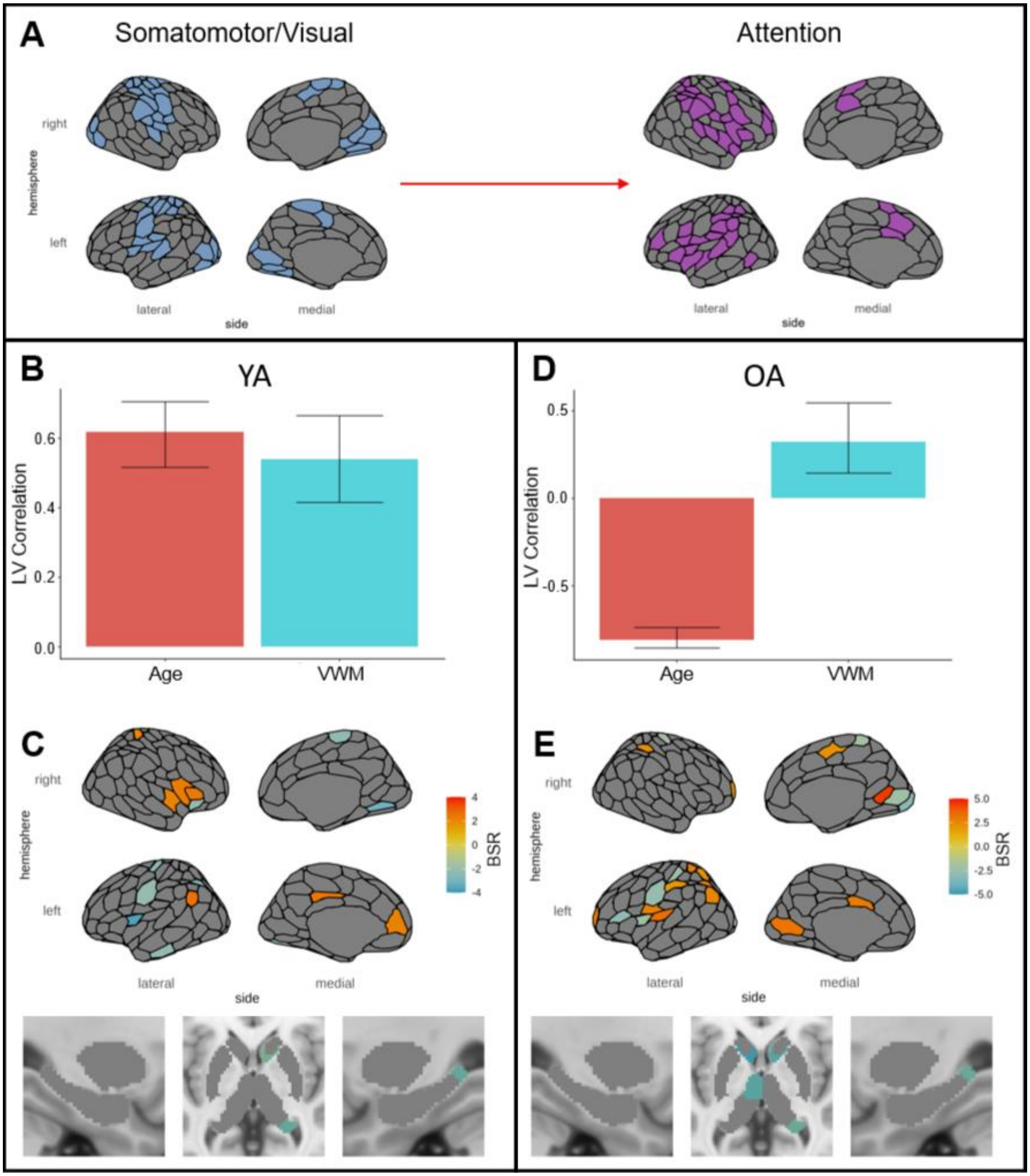

Supplementary Figure 3. PLS analyses with independent variables of control energies (with ρ = 1.1) for a state transition from the Somatomotor/Visual to the Attention state (**A**) and dependent variables of age and VWM. (**B**) and (**D**) depict the behavioral correlation of age and VWM with the LV, while (**C**) and (**E**) represent the BSRs highlighting the regions with reliable positive associations with age and VWM in yellow to red and the reliable negative associations with age and VWM in green to blue.

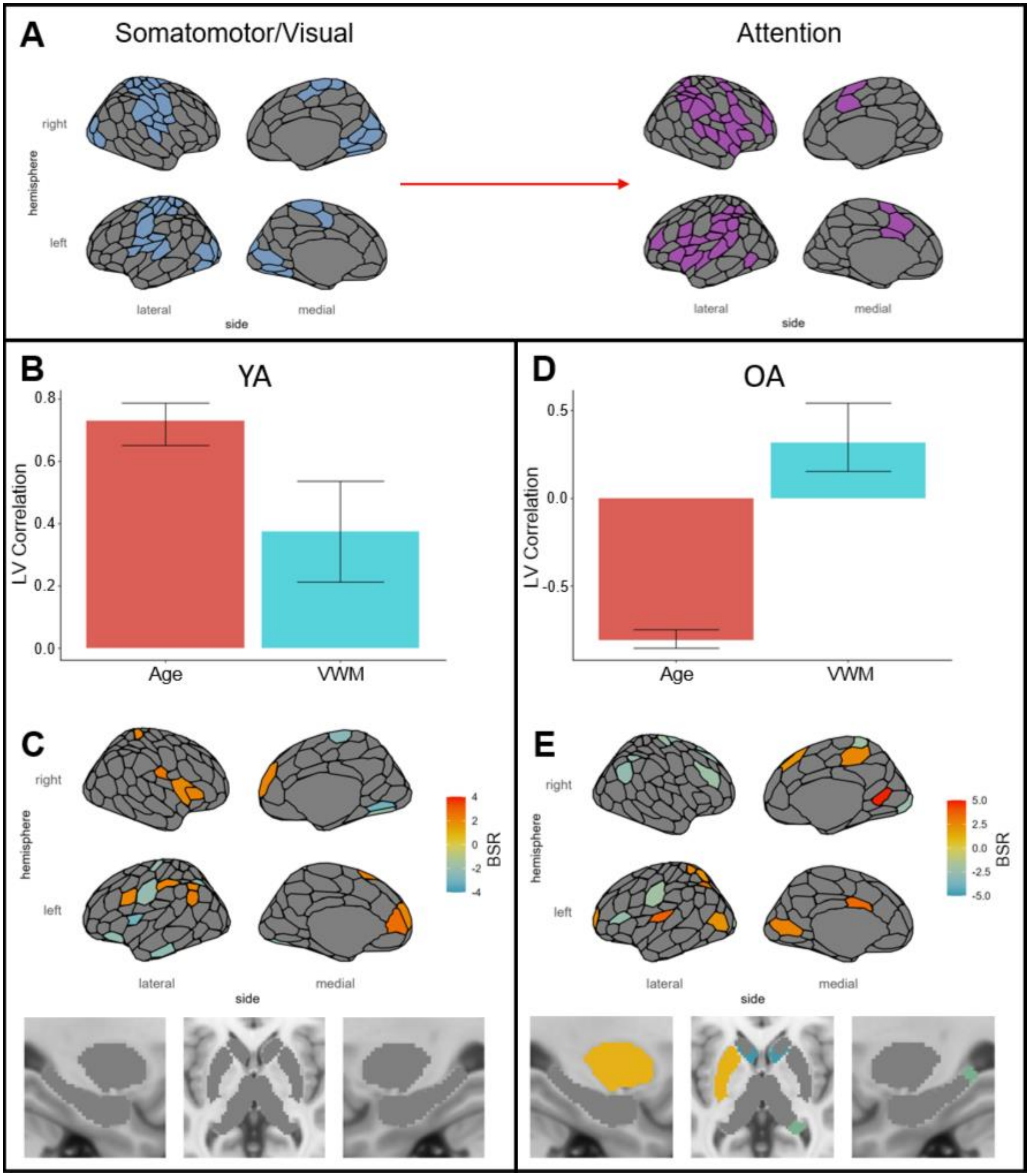

Supplementary Figure 4. PLS analyses with independent variables of control energies (with ρ = 0.1) for a state transition from the Somatomotor/Visual to the Attention state (**A**) and dependent variables of age and VWM. (**B**) and (**D**) depict the behavioral correlation of age and VWM with the LV, while (**C**) and (**E**) represent the BSRs highlighting the regions with reliable positive associations with age and VWM in yellow to red and the reliable negative associations with age and VWM in green to blue.

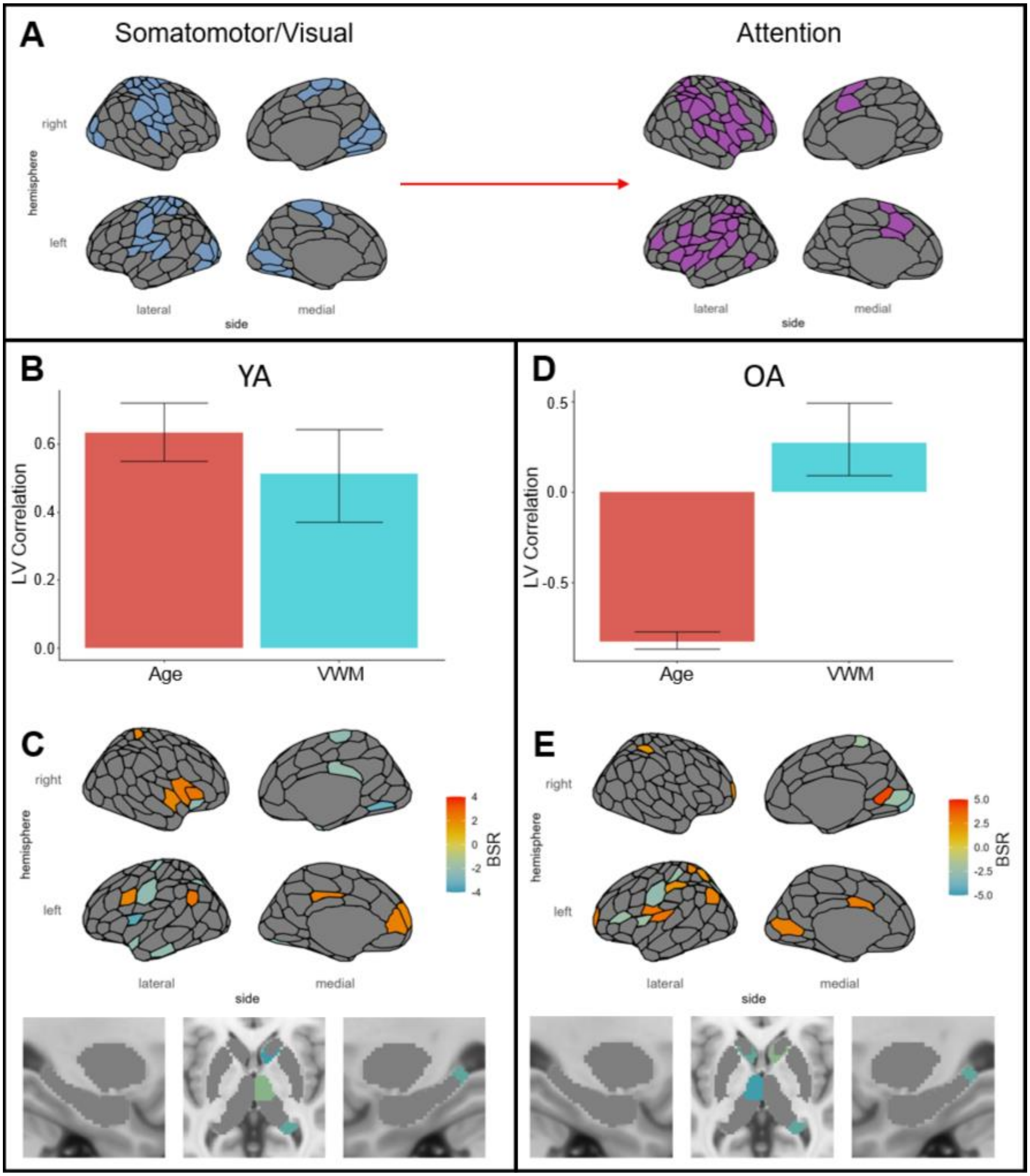

Supplementary Figure 5. PLS analyses with independent variables of control energies (with ρ = 10) for a state transition from the Somatomotor/Visual to the Attention state (**A**) and dependent variables of age and VWM. (**B**) and (**D**) depict the behavioral correlation of age and VWM with the LV, while (**C**) and (**E**) represent the BSRs highlighting the regions with reliable positive associations with age and VWM in yellow to red and the reliable negative associations with age and VWM in green to blue.

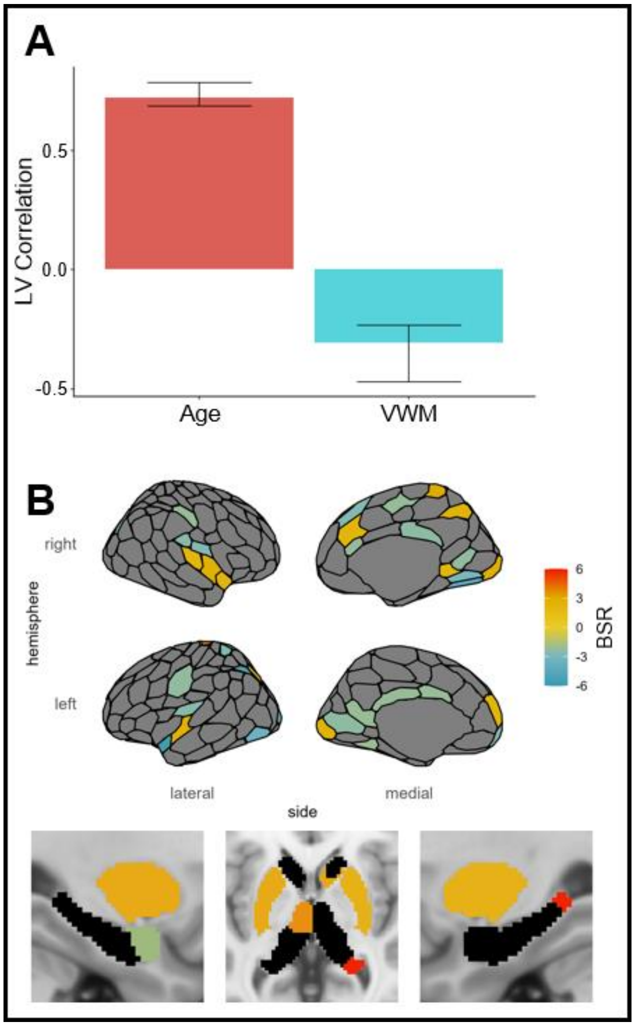

Supplementary Figure 6. PLS analyses (all ages included) with independent variables of control energies for a state transition from the Somatomotor/Visual to the Attention state and dependent variables of age and VWM. (**A**) depicts the behavioral correlation of age and VWM with the LV, while (**B**) represents the BSRs highlighting the regions with reliable positive associations with age and VWM in yellow to red and the reliable negative associations with age and VWM in green to blue.
